# Decoupling touch from sex: gender(ed) representations of physical intimacy in the cuddle industry

**DOI:** 10.3389/fsoc.2023.998037

**Published:** 2023-07-27

**Authors:** Paulo Santos

**Affiliations:** IRCCS San Camillo Hospital, Venice Lido, Italy

**Keywords:** intimacy, sexuality, body work, gender representations, professional cuddling

## Abstract

The present study explores the resistance to and potential transformation of hegemonic gender norms regarding intimacy and sexuality through an instrumental case study of the so-called “professional cuddlers”, a category of body workers that have proliferated in Europe since 2015 offering paid sessions of non-sexual physical intimacy. Despite efforts to frame the service as a form of therapy, professional cuddling is often misunderstood as a front for prostitution, and practitioners must frequently deal with unwanted sexual advancements. Drawing from a 3-year online ethnographic study of cuddling services, the dataset includes 10 in-depth interviews informed by a previous qualitative analysis of 46 newspaper articles, 16 forum discussions, and 25 websites related to such practices. Findings demonstrate that the representational limbo experienced by practitioners could be better understood as a product of the “sexusociety”. Though it is unclear whether professional cuddling has any significant impact on hegemonic gender norms, results show that it nonetheless deconstructs the normative landscape through the enactment of alternative scripts.

## 1. Introduction

People are nowadays resorting to the market to mitigate the impact of three important factors that have deeply transformed our society: migration to urban areas, the ascent of the working woman and the rise of divorce rate (Hochschild, 2012). With the “intellectualization” of daily life and the transformation of intimate relationships into cognitive objects that can be exchanged (Illouz, 2007), the bulk of care and sex work that used to be carried out for love is now being done for profit (McDowell, 2009). This growing tendency, as suggested previously, has been facilitated by the social changes that industrialization and capitalism have engendered, such as the erosion of community and family life. Under these circumstances, we have witnessed a proliferation of private businesses that surrogate social practices once confined to the household or the neighborhood, thus fostering a new “commodity frontier” (Hochschild, 2003). Regardless of the “hostile worlds” view that conceives the intimate and the economic as two fundamentally incompatible spheres due to the destructive consequences that the latter may have over the former, it seems that these commodified forms of intimacy have increasingly gained cultural acceptance (Zelizer, 2005). In Europe, for instance, there is a particularly high market demand for caring and sex services that are mostly provided by migrant women (Agustín, 2007).

It is against such backdrop that professional cuddling emerged, presenting itself as a therapeutical practice which enables both recipients and practitioners to experience a nurturing and relaxing session of mutual non-sexual touch. However, despite the efforts of professional cuddlers to desexualize their line of work, media and public opinion often end up depicting cuddling services as a front for prostitution. Consequently, practitioners experience a stigmatizing representational limbo regarding their professional stance.

The spectrum of services and practices a professional cuddler may offer is somewhat broad but sessions typically consist in providing clients with hugs, snuggles and massages within a safe environment, intended to be free of bias, conflict, criticism, or potentially threatening actions, ideas or conversations. Sessions will often take place at the practitioner's studio, commonly located near other wellness experts such as holistic healers, chiropractors, massage therapists and yoga teachers. Studios are minimal yet cozy in their decoration with dim, warm lights matched with scented candles and relaxing, ambient music setting the atmosphere. Several cushions, blankets and futon mattresses are normally available for clients to feel comfortable in whatever cuddling position they choose to settle (face hugging, head on lap, lounge chair, spooning). Less typical, though not uncommon, services that some practitioners offer as well are watching movies, preparing meals together, walking in the park while holding hands and other companionship activities. In the absence of a personal studio, professional cuddlers who prefer not to bring clients home, will conduct sessions at hotel rooms or at the client's own quarters.

What is so unique about professional cuddling, is that it does, indeed, provide similar comforts that many sex workers offer to their clients, except that it allows no space for sexual achievements. It may (and it does) sound surprising for many, but the idea according to which men who purchase sex are simply seeking to satisfy a biological need (i.e., ejaculation) is a common misconception. After all, research on the sex market has shown that many customers pursue not only physical, but also social and emotional satisfaction (Sanders, 2008). Moreover, physical intimacy doesn't necessarily have to be sexual in nature nor does it have to come in the form of an orgasmic release, in case it is. In fact, it is not unusual for sex workers to solely provide intimate non-sexual physical interactions (Sanders, 2007).

At times, cuddling services might resemble the so-called *girlfriend experience*, a service commonly provided by middle-class sex workers in which the escort simulates being a girlfriend through the engagement of emotions and affects which are usually limited to the private sphere, thus forming an “authentic, yet bounded, interpersonal connection” (Bernstein, 2007; p. 474). According to the professional cuddlers that were interviewed for the present study, this could be particularly true in those cases where practitioners concede, upon customer request, to perform activities that are more akin to companionship services than to what professional cuddling is actually supposed to provide. However, many practitioners choose to distance themselves from these requests because, as they argue, it blurs the boundaries between work and private life, which in turn raises concerns about the potential development of romantic feelings.

All in all, professional cuddlers maintain that what they offer is a non-sexual and non-romantic service, but not everyone shares that same conviction. As it has been pointed out by other body workers who often deal with stigmatizing representations of their professional identity and that experience similar tensions (Wolkowitz, 2002), many are skeptical about what happens behind closed doors. These are commonly held opinions that can be observed at online forum discussions as well as media articles about cuddling services. In fact, there is a certain inclination toward focusing on the sexual rather than therapeutic aspects of the practice, which is not so surprising given that “body work involves confronting the sexual meanings of the body and, therefore, always involves sexualization/desexualization” (Cohen et al., 2013; p. 10). Massage therapists, who have a long history dealing with wrong perceptions regarding their occupation (Oerton and Phoenix, 2001; Sullivan, 2012), are an emblematic example. For this reason, several efforts have been made to desexualize the practice (Sullivan, 2014) by deploying certain “discursive formations or professional identifications” (Oerton, 2004; p. 553) that purge their image from the “dirty work” label which is so often associated to physically intimate labor (Simpson et al., 2012). Much like massage practitioners set boundaries to separate massage therapy from sex work, so do professional cuddlers. Except that for the latter, the need for desexualization strategies is greatly exacerbated not only by the subjects' gender, given that it plays an important role on how intimate interactions are socially perceived and understood (Simpson, 2009), but also by the fact that, since recipients are encouraged to cuddle back practitioners, there is a less clear distinction between the body that is working and the body that is being worked upon.

As Sullivan has suggested, “gendered tensions in the professions are revealed when the impetus to monitor and control sexuality rests on individuals. Overall, women must properly control their bodies and men must maintain their status as ‘masculine”' (2014; p. 349). It is not a coincidence that men who perform feminized labor are frequently seen as predators or homosexuals (Lupton, 2000; Sargent, 2000). These assumptions are determined by an underlying traditional principle according to which masculinity and intimacy don't blend because male touch carries a sexualized meaning, especially within more intimate contexts (Fisher, 2009).

To date, sociological literature on professional cuddling seems quite limited. It has been used as a case study to explore how non-normative intimacies raise anxieties and hostilities that “illuminate various facets of normalization and their role in the regulation of affect” (Szegheo-Lang, 2015; p. 21) or how boundaries are negotiated in similar settings (Mayr, 2023). With the present study though, it is my aim to explore how professional cuddling sessions, albeit structured around hegemonic understandings of masculinity (Connell and Messerschmidt, 2005), still have the potential to resist (and perhaps transform) gender norms through the enactment of alternative scripts.

On one hand, desexualization strategies incur in the risk of reinforcing those same social representations which they are up against, precisely because of being rooted on the idea that sexuality at work is dangerous. In other words, it is as if the only possible manifestation is either through harassment or coerciveness, where men are aggressors and women are victims hence reproducing the same old socio-cultural beliefs regarding gender behavior that strip individuals from their subjectivity. This does not mean that sexual advancements aren't a real issue for body workers in general. As the famous *Thomas theorem* posits, “if men define situations as real, they are real in their consequences” (Thomas and Thomas, 1928; p. 572). Therefore, many professional cuddlers express concerns about recipients who misunderstand the scope of what the service offers, which is also one of the reasons why so much attention is paid to the practitioner's self-presentation, to the professionalization of the service and to the screenings that are performed on customers. On the other hand though, the particular ways in which professional cuddlers frame physical intimacy and address sexuality whenever it arises, propose a different picture where mutual touch and caressing don't necessarily have to convey an implicit sexual desire. But even when it does, they enable both parties involved in the session to express such desire and choose sexual inactivity, thus liberating them from the pressures of conforming to sexual norms (Hooks, 2000).

## 2. Methodology

### 2.1. Online ethnography

Conducting an online ethnography was a methodological choice related to two specific reasons. Firstly, because professional cuddling is a multi-sited phenomenon, a condition that posed considerable geographical obstacles since the research was conducted from Italy between 2017 and 2019, and then from Portugal between 2019 and 2020. Back then there were no professional cuddlers in either of these two countries. Secondly, because most of the daily tasks carried out by professional cuddlers are mediated by technological devices (i.e., computers, smartphones, tablets) and often take place on the Internet. To this matter, Hine (2015; p. 32) argues that ethnographic research should consider the blended nature of late-modern societies, a notion that is well illustrated by her *E*3 *Internet* metaphor:

For development of an ethnographic strategy for the Internet, it has seemed particularly significant that it is embedded in various contextualizing frameworks, institutions, and devices, that the experience of using it is embodied and hence highly personal and that it is everyday, often treated as an unremarkable and mundane infrastructure rather than something that people talk about in itself unless something significant goes wrong.

Just like many other forms of body work, besides being part of the informal sector, professional cuddling is also fairly invisible as it is performed one-on-one, behind closed doors, and frequently outside of the conventional notion of the workplace, such as at the practitioner's or recipient's home. For this reason, and considering the elevated costs of a cuddle session as well, observation was limited to the virtual environments in which professional cuddlers operate their businesses and interact with the public. Some examples are the several cuddling agencies and cuddling social networks that can be found online, where it is possible to observe not only how stakeholders articulate information to present the service according to a specific frame, but also the interactions between community members at the discussion forum. Other examples are the newspaper articles where practitioners get interviewed or certain sub forums from Reddit in which many professional cuddlers have disclosed information about their occupation to a wider audience of users that have the chance to interact with the interviewee.^1^

### 2.2. Field definition

Resonating with Hammersley and Atkinson (1983; p. 32) argument that the definition of settings isn't natural since “boundaries are not fixed but shift across occasions [...] through processes of redefinition and negotiation,” Burrell (2009; p. 182) proposes that, ultimately, the field site is “the stage on which the social processes under study take place” and that it is “constructed rather than discovered” through a process “of exclusion and inclusion.” Thus, efforts were made to patch each virtual terrain into a network of “interconnected (web) sites and their communities of users” (Airoldi, 2018; p. 3) by adopting some modes of construction outlined by Marcus (1995) for approaching multi-sited fieldwork: *following the metaphor, following the conflict* and *following the people*.

### 2.3. Empirical framework and research questions

The empirical dataset from which this research draws its results is the product of a 3-year online ethnographic study of professional cuddlers and their social world, consisting in a combination of several iterative rounds of data collection, with each stage informing the next. During the exploratory stage, observations—though mostly non-participant—were made overtly on the community's own forums and covertly on other public forums not requiring access credentials. A total amount of 46 newspaper articles, 16 forum discussions and 25 websites related to professional cuddling were collected and analyzed along with Nonhoff (2017) principle of “discourse analysis as critique”. This first stage helped formulating a hypothesis (H) which, in turn, developed into a more specific set of research questions (RQ):

H. Professional cuddling is often perceived as sex work due to a general conflation of intimacy and sexuality, whereas the latter is considered to be a prerequisite to the former.

RQ1. Why is professional cuddling often confused with sex work?

RQ2. What mechanisms underpin such representations?

RQ3. How do professional cuddlers manage their identity and tackle the stigma associated to their line of work?

Once the problem was defined, besides having exchanged e-mail correspondence with some relevant actors and analyzed documents related to cuddling services, formal in-depth interviews with professional cuddlers were conducted according to a semi-structured script consisting in open-ended questions, or rather prompts^2^, that addressed (a) the process of becoming a professional cuddler, (b) the aspects involved in being one, (c) how sessions are conducted and managed, and (d) the perceptions regarding public opinion and media depictions of cuddling services. All these dimensions had emerged in the course of the exploratory stage.

Interviewees were recruited according to a heterogenous purposive sampling (also known as judgment sampling) technique which is particularly useful for studying new cultural domains through the knowledge provided by the experts that can be found within. This technique is not oriented toward randomization nor probability, instead the researcher deliberately chooses key informants (Bernard, 2002) who have a deep knowledge of the culture being studied (Campbell, 1955; Tremblay, 1957; Seidler, 1974), based on what needs to be known. In this case, the goal was to obtain an insider perspective about cuddling services from a diversified point of view. Hence, an attempt was made to recruit professional cuddlers with different profiles (age, gender and nationality).

The study focused mainly on the emergence and development of cuddling services in Europe which, despite having first appeared in the UK around 2012, only burgeoned between 2017 and 2018. Considering that it was (and still is) an emergent niche market, research participants were hard to recruit for several reasons: timezone differences, language barriers and number of available practitioners working within European territory. Out of the 10 practitioners that accepted to be interviewed, 4 worked from Germany, 3 from the UK, 2 from the Netherlands and 1 from Switzerland. Ages ranged from 25 to 55 years old and, although the occupation is largely performed by women, after many fruitless efforts to recruit male participants, one man finally conceded to be interviewed.

Informed consent was obtained from all participants and all names were pseudonymized in order to comply with the principle of “data minimization,” expressed in Article 5(1)(c) of the GDPR and Article 4(1)(c) of Regulation (EU) 2018/1725, which states that personal data must be “adequate, relevant and limited to what is necessary in relation to the purposes for which they are processed.” Reddit usernames were left in their original form since, contrarily to most social networks, the platform doesn't require any personal information such as a real name or a profile picture. Additionally, all subreddits, threads, posts and comments on Reddit are available without any access restriction and/or registration being required, making it public information and naturally occurring data. Finally, given that many official ethics guidelines recommend disguising identities as a default position (see, for example, British Sociological Association, 2017), names and addresses of cuddling agencies and community websites were omitted.

## 3. The social world of professional cuddlers

The idea that social worlds are not just “forms of communication, symbolization, [or] universes of discourses” (Strauss, 1978; p. 120), which were Mead (1972) main focus of attention, sends us back to the symbolic interactionist tradition rooted in the Chicago School's ecological approaches to social phenomena, which lied on “the ability to focus now on the niche and now on the ecosystem which defined it” (Dingwall, 1999; p. 217). As Strauss argued back then, in order to achieve a better understanding of social worlds it is also necessary to “examine palpable matters like activities, memberships, sites, technologies, and organizations” (1978; p. 120). With the growing involvement of symbolic interactionists in Science and Technology Studies, infrastructures came to be gradually incorporated into the framework since they act “as frozen discourses that form avenues between social worlds and into arenas and larger structures” (Clarke and Star, 2008; p. 115).

As suggested by scholars who have developed the previously discussed model, the following sections are dedicated to a brief exploration of the cuddle industry's virtual avenues. Navigating through the social world of professional cuddlers will provide a greater awareness of the meanings associated to the practice and, thus, facilitate the interpretation of the testimonials that shall be discussed later in the text.

### 3.1. Cuddling social networks

From the practitioners point of view, there is no pre-established itinerary toward the world of cuddling services but, from what has been gathered in the course of the present study, cuddling social networks are often a starting point for two specific reasons.

First of all, because it provides immediate visibility within the community and exposure to potential clients since not all users are professionals. To access the platform, just like any other social network, all that is needed is the registration of an account. Once that is done, the user is allowed to personalize his/her profile with several informations (i.e., sexual orientation, marital status, height, body type, ethnicity, religion, job) and search for other people according to those same parameters. Additionally, there is a public forum where users can discuss about all sorts of topics which, as expected, concern mostly the community's practice.

Secondly, because there are fewer requirements that must be fulfilled in order to become a professional cuddler. As stated on one of the platforms that were analyzed, these amount to having “a photo to attach,” being “affectionate to anyone,” “accepting all races, ages, genders and sexual orientations,” being “reliable with strong interpersonal and communication skills,” and finally “understand and agree to the Cuddler Contract.” Among other things, the contract states that:

3. The Cuddler must not offer any non-platonic services (i.e. fetish related, sexual services, etc.) […]

15. No sexual activity is permitted. You are not allowed to solicit for sex [...]

16. Both parties will remain clothed the entire session. Undergarments do not constitute as sufficient clothing. If either party needs to change clothing this will be done in private and out of sight of the other party. [...]

17. No touching in areas covered by undergarments is permitted. [...] No kissing is allowed.

Materializing an agreement is crucial to reduce the “double contingency” (Parsons, 1951) that is here exacerbated by the extraordinary characteristics of cuddling services. The contract is an expression of a symbolic system in which the norms and values that regulate the interaction between professional cuddler and client are shared. It is based on the premise that the actors would otherwise experience an overwhelming loss of certainty regarding the meanings implied by the actions at stake.

Other peculiar features of cuddling social networks that contribute to the “definition of the situation”^3^ (Thomas and Znaniecki, 1927) are the sections in which a sort of statement of purpose is presented. For instance, looking at the “How It Works” section of this same platform, a diachronic comparison of the potential user's life before and after being introduced to the cuddling social network is rendered as follows:

How it Used to Work

You know a friend who would make a great cuddle buddy. You're fine being platonic friends because what you really want is to snuggle up on the sofa and watch a movie.

It makes sense to have friends that you regularly cuddle with, but you struggle to find the courage to ask them because you worry they won't understand.

While feeling very awkward, you eventually suggest the idea but they misinterpret your approach as a romantic proposition.

There's now too much doubt over the sincerity of your friendship. It's never quite the same again.

How it Works

You sign up for free in under 30 seconds. You can then instantly communicate without any barriers—it's completely free.

You search for people in your area out of the thousands of active members who are looking to cuddle. We invented online cuddling so you'll find more choice here than anywhere else.

You send them a message but you won't need to worry about how to word it—they already get it. They're here for the same reason you are; to cuddle and nothing more.

They sound like someone you'd get along with, so you organize to meet-up and cuddle. And most importantly, it's pressure-free without any expectations of it progressing to something more.

These two items provide a (non-)normative frame that subverts traditional expectations regarding intimacy and sexuality. They reflect not only the presence of a dominant social order where physical intimacy is understood as sexual and/or romantic, but also the existence of individuals that stand at the opposite pole by not conforming to those same “taken for granted” (Schütz, 1944) assumptions. Sections like the one shown previously, are an invitation for new members to adopt “a different scheme of interpretation for the meaning of an act” (Goffman, 1971; p. 231), thus reinforcing (or compensating for) what will then be metacommunicated during the cuddle session, in Bateson's own words, that “these actions, in which we now engage, do not denote what would be denoted by those actions which these actions denote” (Bateson, 1972; p. 180).

Last, but not least, on the “Terms and Conditions” section, the user can find further contextual information regarding the platform and its rules:

You agree to never use this Website for the intent of meeting another member for sex. You also agree to never attempt to progress a meeting, organized via this website, to a sexual nature.

When communicating with another member, you agree to never indicate a desire to cuddle while doing any of the following: (1) being nude, (2) wearing only underwear, (3) kissing, (4) groping, (5) satisfying a fetish or kink, and (6) anything of a non-platonic nature. [...]

This site is intended for platonic services only. Members or professional cuddlers attempting to solicit or offer non-platonic services (i.e. fetish related, sexual services, etc.) [...] will be banned. This is to further keep the cuddling community image separate from any illegal activities [...]

You agree to only upload photos of yourself that are fully clothed. Your photos cannot be suggestive whatsoever, even to a mild degree (e.g., focus on the cleavage).

The specified terms support the code of conduct that is already encouraged in the aforementioned contract. In addition, they attempt to exert a “frame alignment”^4^ on all potential users in order to create resonance (Snow et al., 1986) with the new social context. As we have seen, cuddling social networks operate under a specific set of rules that temporarily suspends the outsiders' “schemes of interpretation and orientation”^5^ (Schütz, 1962) and, in so doing, it simultaneously enables them to internalize new meanings ascribed to certain social cues that will arise in that particular setting, thus avoiding misinterpretations and providing alternative courses of action. Plus, it creates a “safe space” for those whom already share that same ideal according to which physical intimacy can be experienced and expressed without creating sexual expectations.

### 3.2. Cuddling agencies

In some cases, joining a cuddling agency is the next step that practitioners take toward reaching a more legitimate and professional image regarding their occupation. Just like any other form of association, these agencies provide peer acknowledgment and recognition, which in turn makes the individual feel safe about the social value of his identity (Rawls, 1971; Honneth, 1992) precisely because one's identity is shaped by social recognition. In that sense, associations are safe havens where members support each other's conception of a “good life” and thus find confirmation of their own self-image (Rosenblum, 1998; Warren, 2001).

Cuddling agencies differ significantly from cuddling social networks because they create a sort of enclave. In fact, to join a cuddling agency it is necessary to attend a paid training course provided by that same agency and obtain a certification, which is only granted after having passed a theoretical, and sometimes practical, test. Once these requirements are fulfilled, practitioners will have the possibility to see their profile available on the website.

While cuddling social networks are particularly concerned with desexualizing and deromanticizing physical intimacy, cuddling agencies on the other hand emphasize the health costs and consequences of touch deprivation. As it is stated on the “Why We Need Touch” section of one of the analyzed cuddling agencies:

Because human beings are wired to touch and be touched, the absence of touch causes disturbances in both mind and body. Touch helps create psychological and physical wellness, and touch deprivation contributes to illness at many levels.

1. Stress and Relaxation: Touch deprivation increases stress and body tension levels behaviorally and biochemically. […]

2. Sleep Difficulties: Research has found a strong relationship to touch deprivation and sleep disturbance. […]

3. Immune Response: Touch deprivation can suppress the response of the immune system. […]

4. Delayed Growth: There have been many studies linking touch deprivation and growth deprivation. […]

5. Cardiovascular Disease: Cardiovascular disease is often exacerbated by a lack of contact with other people.

Conversely, the health benefits of cuddling services as a potential solution to the lack of touch are asserted likewise on the “Who Books a CPI Certified Cuddler?” section:

DID YOU KNOW?

Touch triggers a cascade of healing chemical responses including a decrease in stress hormones and an increase in seratonin and dopamine levels. Additionally, touch has been shown to increase the immune system's cytotoxic capacity, thereby helping our body maintain its defenses and decreasing anxiety, depression, hyperactivity, inattention, stress hormones and cortisol levels.

The need to evoke medical discourse, resonates with the “discursive formations and practices deployed by women therapeutic massage practitioners in terms of setting and maintaining professional boundaries” (Oerton, 2004: 550) that separate these occupations from sexual services. Plus, if we assume, as suggested by Bourdieu (1993), that legitimation is based on the audience's belief in the evaluations of a given institutional agent, then any activity that is supported by the scientific community is, in theory, more likely to be acknowledged by the public. In any case, the recurrent conflation between certain types of body work and sex work is perhaps the main driving force that leads practitioners to “have a special and vested interest in being seen as legitimate, serious, healthcare professionals” (Oerton, 2004; p. 550). Indeed, it is not a coincidence that on the “Mission Statement” section the cuddling agency declares its purpose as follows:

To train, inspire, support and unite every Professional Cuddler […]

To maximize your professional credibility and enable your Cuddle Therapy practice to thrive through gold standard training and aftercare. […]

To promote the consensual, non-sexual holding and touching of adults within defined boundaries as a legitimate and valuable holistic therapy, and to remove the stigma and suspicion surrounding the professional provision of platonic touch.

Even if cuddling agencies are, in general, more focused on professionalizing the practice comparing to cuddling social networks, employing desexualization strategies is nonetheless an integrative part of such boundary work. As it has been noted by scholars who have conducted research within the field of body work, “one's professional identity can be considered suspect for many reasons” (Sullivan, 2012; p. 273). In this particular case, which recalls similar issues that concern massage therapists, professional cuddlers adopt medical discourses in order “to manage a sexualized and stigmatized professional identity” (ivi: 274). Differently from massage therapy though, as illustrated by the previous excerpt, professional cuddling agencies explicitly state that the service is non-sexual^6^ and go even further by claiming that the promotion of consensual touch and the removal of stigma surrounding practitioners is part of the community's mission. Such a straightforward definition can also be observed by looking at the “Code of Conduct” section, available at the website of a different cuddling agency:

6. This is a strictly platonic service. Client and Practitioner both agree to not pursue or encourage sexual arousal. Also:

• Minimum clothing of tank top and shorts that cover the top half of the thigh and are suitable to be worn in public for both Client and Practitioner at all times.

• No hand to genital or breast contact. No intentional genital stimulation of any kind.

• No exchanging of saliva, or any other bodily fluid, in any way.

Yet, regardless of all the outspoken and unambiguous disavowals of sexual achievements through professional cuddling, what perhaps distinguishes the practice from other occupations that operate under similar settings and deal with the same sort of situations is the way it addresses the expression of sexual desire. This is made clear on the cuddling agency's “FAQ”:

What happens if I become sexually aroused during a session?

Arousal is a healthy human response to all kinds of things including touch. It is not a problem in a non-sexual session. The important thing is that neither client or practitioner respond to it in a manner intended to increase arousal. It can be acknowledged or not and simply allowed to come and go without taking focus.

Such attitude points out to the recognition of “sexual agency in response to sexualized interactions” and acknowledges a form of “*professional sexuality* that can exist between the (constructed) poles of desexualization and re-eroticization.” (Sullivan, 2014; p. 359).

It should be taken into consideration that, since most practitioners are women and recipients are men, the enacted neutralization of sexual desire can potentially trigger a reconsideration of gender norms. In other words, by “asserting their right to choose, women challenge the assumption that female sexuality exists to serve the sexual needs of men” (Hooks, 2000; p. 157), while men go beyond “gender stereotypes that distance [them] from physical nurturing” (Harding et al., 2008; p. 89). By validating “mutuality as a guiding principle in their sexual negotiation” (Cense et al., 2018; p. 287), men also challenge dominant notions of masculinity as incompatible with platonic intimacy due to the supposedly sexualized nature of male touch.

### 3.3. Media representations of, and online discussions about, cuddling services

Professional cuddling holds a promising potential toward changing the gender normative landscape. However, such possibilities are nonetheless undermined by press depictions that build on, and thus exacerbate, the assumption that physical intimacy and sexuality are two sides of the same coin. In fact, the most recurrent topic throughout the media is sex, or rather how the service is (surprisingly) non-sexual. As illustrated by Armour (2015) in an article published on The Wall Street Journal:

For $80 an hour, or up to $400 for an overnight gig, the 33-year-old mother of three dons flannel pajama bottoms, puts away her family pictures and two pit bull mix dogs and invites clients into her bedroom in Highland, N.Y., to snuggle. Once the spooning begins, she insists that it stay strictly platonic. The cuddle-for-hire business is taking off—even though the clothes stay on.

Why does she have to insist that professional cuddling is a *non*-sexual service? And why is it so striking that although the clothes *stay on*, the business still *takes off* ? Despite recent claims that media articles “have been sure to distance [professional cuddlers'] *non-sexual* economic exchanges from pretty much any and all forms of sex work” (Szegheo-Lang, 2015; p. 18), such demarcation is often elaborated with skeptical undertones. Upon closer look, it becomes evident that the underlying premise predicts that along with physical intimacy, sexuality must necessarily emerge. This same attitude finds confirmation on several online discussions about cuddling services. The following excerpts, which have been extracted from a *subreddit* called “AskMen,” reflect how some users reacted to a thread posted by a woman nicknamed (Cuddlme, 2013), who claimed that she would soon become a professional cuddler but was curious to know why there was “some hatin' going on […] about this service”:

Why would I pay someone to cuddle when I could just pay a prostitute to cuddle and I could get a blowjob at the end? (deleted user)

Simply steel yourself for two likely scenarios:

-some costumers will misunderstand the concept and ask for a little extra;

-some will become attached, ask for your phone number etc. (cccjfs)

Cuddling is about physical intimacy. I guarantee you, you will have a few boners a day rubbed into your back. (nubbeh123)

Hope you like getting poked with boners while being spooned. (vbfronkis)

Generally speaking, the reactions were quite harsh and demeaning. Users expressed several motivations as to why professional cuddling is “hated” but, among most, the nexus between physical intimacy and sexuality appeared to be central. For instance, the first user fails to understand the whole point to cuddling if it is stripped from sexual satisfaction, while the remnant comments warn the practitioner that male clients will experience sexual arousal. These representations of male behavior within physically intimate settings reinforce the ready-made sexual scripts that are endorsed by traditional gender norms. They reproduce the so-called “sexual double standard” according to which “men are expected to present themselves as sexually active and ready to take sexual initiatives, whereas women are expected to present themselves as sexually reactive and passive” (Emmerink et al., 2016; p. 363).

## 4. Cuddling services from the inside out

These are some of the social pressures that undermine the potentials of professional cuddling as a legitimate practice. The constant focus on the sexual dimensions of a service that, as it has been shown previously, struggles to present itself as non-sexual, is an indicator of how the “sexual imperative” (Przybylo, 2014) structures social understandings of physical intimacy around sex. Cuddling is thus perceived as a preliminary and integrative part of the sexual act. If it does not lead to that, then it is not only pointless but also unbelievable. Emma, a 33-year-old cuddling agency owner from Germany who began as a solo professional after graduating in German Literature and Philosophy with a thesis on “body intelligence,” briefly explains how she usually experiences speaking about her services to the media:

I did some interviews on the radio, television and for newspapers as well and the first questions they ask you are: “is it something sexual?,” “is it naked?,” “do men attack you?,” “do they get aroused?” [...] I think that when people first hear “cuddling services” they think it's a euphemism for sexual services.

Another common feature of the discourses that surround professional cuddling, as suggested in the previous excerpt, is the depiction of men as aggressors and pursuers of sex. Questions such as “do men attack you?” and “do they get aroused?” validate and reproduce traditional gender stereotypes based on the “‘male sexual drive' discourse [which] sees men as sexually insatiable and male sexuality as naturally an uncontrollable drive.” (Hollway, 1984; p. 63). In fact, it is not a coincidence that professional cuddlers set up several boundary-building stages prior to the session such as vetting calls to filter right from wrong customers or agreements that require a signature. Lydia, a 35-year-old professional cuddler from Czech Republic who moved to England some years ago to pursue a nursing career, explains how she proceeds after being booked:

I just arrange for a video call and we'll just discuss- I just go through... I just ask them why they booked a session and what do they expect from it... What... What are they looking for... Because this is important to me, I need to know whether they're really happy that it's platonic, so I make sure that they are aware it's only platonic.

Vetting calls are instrumental to professional cuddlers because they allow them to screen customers and learn about their motivations and expectations. As Lydia pointed out, she must know if they are aware that the service is non-sexual. Regardless of the customer's answer though, it is quite common for practitioners to explain the code of conduct over the phone. Finally, once the boundaries of the session have been clearly laid out, the practitioner prompts the customer with a contract stating that he/she agrees with the previously discussed rules. Rita, a 51-year-old mother who was born and raised in England where she also works as a “laughter yoga” teacher, illustrates the procedure as follows:

The client arrives, we introduce ourselves, we go once again through what the session might entail [...] right at the beginning of the session I'll ask my clients to sign a cuddle contract and it's very short and... What it says is everything that we've already discussed in our conversations so that it's not a sexual service […] and that we must be fully clothed […] So, all the things that will keep me safe and my client safe. I get them to sign it so that, you know... Just to double check that they're aware.

While the media and the public draw on discourses of “compulsory sexuality” (Gupta, 2015) that privilege certain ways of doing intimacy (i.e. sexual intimacy), professional cuddlers are pushed toward the adoption of desexualization strategies that reclaim the legitimacy of platonic intimacy. Except that, in the process of doing so, they are also perpetuating dominant representations of men as predators and acknowledging “the obsessive repetition of sexual deeds, desires [and] thoughts” that form the “sexusociety” (Przybylo, 2011; p. 448). Which is not so surprising given that, even if the practice clearly acts as a resistance to, it is still embedded in a culture dominated by patriarchy. This also explains why so many professional cuddlers have a hard time finding acceptance among close peers. When speaking about how she felt in terms of telling others about her occupation, Deborah who is 25 years old and, besides working as a professional cuddler herself, runs a cuddling agency in England, said that “it was quite difficult to share in the beginning because I was afraid of how people would perceive me, how they would judge me [...] I was a little bit apprehensive but I took my time.” These concerns are quite common among practitioners and they aren't unfounded since prejudice is right around the corner. Hugo, a 54-year-old divorced Swiss, is one of the few men who work as professional cuddlers in Europe. However, becoming a male practitioner in a female dominated occupation forced him to reconsider his friendships:

I found out that my circle of friends is changing just because of that, because most of my old friends don't understand why I do this. They think that this is not something male, you know? This is very female or they think, as I said, that it has a lot to do with sex. I've explained it has nothing to do with sex but I feel like the society as a whole is not really ready yet for this sort of concept. [...] Society accepts that women are more into that than men because it's considered to be a soft... Profession, right?

For his old friends, he wasn't fulfilling the role and behavior that is prescribed by the dominant notion of a masculinity. Unlike other men who end up succumbing to social pressures, Hugo chose not to conform to traditional gender norms. But this is not simply a matter of gender segregation, as it also implies gendered assumptions regarding sexual scripts. In fact, Hugo has exemplified this by claiming that “when I tell my friends I'm a cuddler […] they don't understand because they say cuddling is something very personal and you only cuddle like foreplay, before sex.” Hugo's old friends perceive him as less masculine not only because of the supposedly feminine qualities of his job but also because, contrarily to what would be expected, he believes that sex isn't necessarily a prerequisite for intimacy. Such expectation found confirmation among the female practitioners' own representations of the male subject:

I don't think they could understand or imagine that if a guy is cuddling with a girl, that the guy doesn't want anything more than just to cuddle with the girl. I think that, specially in the mindset of a man, he cannot help it because that's also how we are, you know? How the system works and how we are raised and how our patterns are... Cuddling... Is a way to get something else. [...] For men to touch is... Often... Through that system. It's socially seen as sexual, you know? If a guy hugs a girl too long or he hugs a kid too long, people maybe think things of it. [...] It's almost expected of them that if they cuddle somebody, something... They have something else on their mind... And it isn't necessarily there but, because the whole world thinks it, it will be there.

As suggested by Karen, a 36-year-old professional cuddler from the Netherlands, men aren't capable of decoupling touch from sex because their “mindset” is guided by the (gendered) “patterns” that the (patriarchal) “system” imposes. When they do, as Hugo's case has demonstrated, they are stigmatized for deviating from the prescribed gender norms and expressing alternative forms of masculinity. Drawing on Rubin (1984) “hierarchical system of sexual value,” Przybylo argues that the sexusociety's favored repetitions are “coital sex, sex with a purpose (be it reproduction or male orgasm), heterosexual and heteronormative sex, sex within marriage or coupledom [...] and a sexuality that amounts to little more than the sum of these” (Przybylo, 2011; p. 448). At this point, it would be useful to consider that such repetitions aren't just limited to sex since they encompass intimacy as well. Therefore, the list of repetitions could benefit from a slight twist: *sexual intimacy, intimacy with a purpose (be it sexual or romantic), heterosexual and heteronormative intimacy, intimacy within marriage or coupledom and an intimacy that amounts to little more than the sum of these*.

## 5. Discussion

It is unclear whether professional cuddling has any significant impact on the normative landscape that governs gendered assumptions about physical intimacy. What is evident though, is that the practice stands in opposition to the gender performances promoted by patriarchal norms (i.e., hegemonic masculinity and emphasized femininity). Indeed, the testimonials and experiences of practitioners, which stand in stark contrast with media and public representations of physical intimacy, show that there are alternative—though perhaps still subordinated—scripts available for both male and female subjects. Despite social disapproval, it seems that subjects who are involved in professional cuddling, actively deconstruct the “sacrosanct trilogy of sex, gender and desire” which “push our lives toward [sexnormative^7^] visions of love, care, kinship and intimacy.” (Oliveira et al., 2014; p. 49). Similarly to what feminist scholars have claimed about asexuality, professional cuddling might as well free women “from authoritarian constraints of patriarchy, demands of men, and less worthy pursuits of pleasure and physical gratification.” (Fahs, 2010; p. 452). This applies not only for women, but for men likewise since masculinity, though dominated by a specific set of stereotypes, is nonetheless plural and in need of emancipation from its harmful hegemonic notions.

To conclude, although light has been shed on some of the reasons why professional cuddling is often confused with sex work—or, in other words, why touch (i.e. physical intimacy) is so intrinsically conflated with sex—and how practitioners manage their identity and tackle stigma, further research would be necessary to fully understand how and whether such practice actually is transformative in terms of gender performances and sexual scripts or not. What can be argued though is that, contrarily to asexuals who have no particular interest in politicizing their identity^8^ (Dawson et al., 2018) as a form of resistance against the “diluted omnipresence of sexuality in our western contemporary present” (Przybylo, 2011; p. 446), professional cuddlers appear to be much more actively concerned with sexualized understandings of physical intimacy precisely because they are directly confronted with such meanings in the course of their practice, on a regular basis.

## Data availability statement

The raw data supporting the conclusions of this article will be made available by the authors, without undue reservation.

## Ethics statement

Ethical review and approval was not required for the study on human participants in accordance with the local legislation and institutional requirements. The patients/participants provided their written informed consent to participate in this study. Written informed consent was obtained from the individual(s) for the publication of any potentially identifiable images or data included in this article.

## Author contributions

The author confirms being the sole contributor of this work and has approved it for publication.

## Footnotes

^1^In this case, sub forums dedicated to AMAs (Ask Me Anything) were particularly useful to see the reactions that the practice triggers on the public.

^2^The aim was to keep the interview fluid enough so that participants could bring up and discuss other topics that they considered relevant (Reinharz and Davidman, 1992).

^3^As the Chicago sociologists explain, “the definition of the situation is necessary preliminary to any act of the will, for in given conditions and with a given set of attitudes an indefinite plurality of actions is possible, and one definite action can appear only if these conditions are selected, interpreted, and combined in a determined way” (Thomas and Znaniecki, 1927; p. 68). In other words, the meaning of a situation is constructed by the individual's wishes, experiences, needs, beliefs and customs of his or her own social group. Consequently, his/her actions are more or less determined by the subjective meaning attributed to the situation in which he/she finds him/herself, rather than by its objective aspects.

^4^Though the concept is originally used by the authors to describe how social mobilization and movement participation occurs, it is useful here to explain a similar process that potentially arises whenever new members join the cuddling community. Drawing on Goffman's notion of “frameworks” (Goffman, 1974), Snow and his colleagues develop the idea according to which individuals and the social movement organizations they choose to associate with share the same “interpretative orientations” because their beliefs, interests and values are, or become, linked to the organizations' ideology, goals and activities (Snow et al., 1986). Similarly, when someone joins a cuddling social network, as in this case, he/she theoretically adheres to the norms that govern action within that specific social realm.

^5^According to Schütz, ordinary social life is governed by typifications formed by knowledge acquired in a given social world through its members, who use these same types as schemes to produce their own actions and understand the actions of others as well (Schütz, 1962). Moreover, these typifications are usually more standardized when it comes to patterns of conduct that have been greatly institutionalized. This interactional dynamic is also reflected by Garfinkel's rework of “the documentary method of interpretation” (Garfinkel, 1967). For the ethnomethodologist, these typifications are a “commonsense knowledge of social structures” that helps actors in their daily reasoning about the social world. Much like a feedback loop, when confronted with certain events or behaviors, actors employ their known patterns as a source of explanation while seeing those same events or behaviors as a “document” that confirms the patterns that they have just used. Hence, “the objective reality of social facts [is] an ongoing accomplishment of the concerted activities of daily life” (Garfinkel, 1967; p. vii).

^6^A recent study suggests that “massage therapy clinics and colleges could display information at their front desks about their zero-tolerance policy for sexual harassment by clients.” (Richard et al., 2020; p. 210) According to the authors, this would serve as a deterrent against potential offenders. Being proposed as a preventative measure, it is here assumed that such desexualization strategy isn't commonly used.

^7^As Chasin argues, “sexualnormativity positions sexuality as the universal and privileged way of being, which is both normalized and socially supported” (Chasin, 2011; p. 719).

^8^Since asexuality is defined by some asexuals as a “lack of something” which not always requires a “coming out,” activism is sometimes seen as rather unnecessary. This is even more the case for “heteroromantic asexual people” who claim that they can “‘pass' quite effectively at an everyday level” (Dawson et al., 2018; p. 14).

## References

[B1] Agustín L. M. (2007). Sex at the Margins: Migration, Labour Markets and the Rescue Industry. London: Zed Books.

[B2] Airoldi M. (2018). Ethnography and the digital fields of social media. Int. J. Soc. Res. Methodol. 21, 661–673. 10.1080/13645579.2018.1465622

[B3] Armour S. (2015). Professional cuddlers embrace more clients. Wall Street Journal. Available online at: https://online.wsj.com/articles/professional-cuddlers-embrace-more-clients-1420759074 (accessed November 18, 2017).

[B4] Bateson G. (1972). Steps to an Ecology of Mind. San Francisco, CA: Chandler Publishing.

[B5] Bernard H. (2002). Research Methods in Anthropology: Qualitative and Quantitative Methods. Walnut Creek: Altamira Press.

[B6] Bernstein E. (2007). Temporarily Yours: Intimacy, Authenticity, and the Commerce of Sex. Chicago, IL: University of Chicago Press.

[B7] Bourdieu P. (1993). The Field of Cultural Production. New York, NY: Columbia University Press.

[B8] British Sociological Association (2017). Statement of Ethical Practice. Available online at: https://www.britsoc.co.uk/media/24310/bsa_statement_of_ethical_practice.pdf (accessed May 23, 2023).

[B9] Burrell J. (2009). The field site as a network: a strategy for locating ethnographic research. Field Methods 21, 181–199. 10.1177/1525822X08329699

[B10] Campbell D. (1955). The informant in quantitative research. Am. J. Sociol. 60, 339–342. 10.1086/221565

[B11] Cense M. Bay-Cheng L. Dijk L. van. (2018). ‘Do I score points if I say “no”?': negotiating sexual boundaries in a changing normative landscape. J. Gender-Based Viol. 2, 277–291. 10.1332/239868018X15266373560443

[B12] Chasin C. J. (2011). Theoretical issues in the study of asexuality. Arch. Sex. Behav. 40, 713–723. 10.1007/s10508-011-9757-x 21541791

[B13] Clarke A. Star S. (2008). “The social worlds framework: a theory/methods packag,”. in The Handbook of Science and Technology Studies (pp. 113–137).

[B14] Cohen R. Hardy K. Sanders T. Wolkowitz C. (2013). The body/sex/work nexus: a critical perspective on body work and sex work. Int. Embod. Sexual. Lab. 4, 3–27. 10.1007/978-1-137-02191-5_1

[B15] Connell R. W. Messerschmidt J. W. (2005). Hegemonic masculinity: rethinking the concept. Gender Soc. 19, 829–859. 10.1177/089124320527863936462930

[B16] Cuddlme (2013). I'm going to become a professional cuddler. I see some hatin' goin on here about this service. What are your thoughts? Reddit. Available online at: https://www.reddit.com/r/AskMen/comments/1ss6h0/im_going_to_become_a_professional_cuddler_i_see/ (accessed March 07, 2018).

[B17] Dawson M. Scott S. McDonnell L. (2018). “‘Asexual” isn't who i am': the politics of asexuality. Sociol. Res. Online 23, 374–391. 10.1177/1360780418757540

[B18] Dingwall R. (1999). “On the non-negotiable in sociological life,” in Qualitative Sociology as Everyday Life, eds B. Glassner and R. Hertz (pp. 215–225). Sage.

[B19] Emmerink P. M. J. van den Eijnden R. J. J. M. Vanwesenbeeck I. ter Bogt T. F. M. (2016). The relationship between endorsement of the sexual double standard and sexual cognitions and emotions. Sex Roles 75, 363–376. 10.1007/s11199-016-0616-z 27688527 PMC5023751

[B20] Fahs B. (2010). Radical refusals: on the anarchist politics of women choosing asexuality. Sexualities 13, 445–461. 10.1177/1363460710370650

[B21] Fisher M. J. (2009). “Being a Chameleon”: labour processes of male nurses performing bodywork. J. Adv. Nurs. 65, 2668–2677. 10.1111/j.1365-2648.2009.05120.x 19824908

[B22] Garfinkel H. (1967). Studies in Ethnomethodology. Englewood Cliffs, NJ: Prentice-Hall.

[B23] Goffman E. (1971). Relations in Public: Microstudies of the Public Order. New York, NJ: Basic Books.

[B24] Goffman E. (1974). Frame Analysis: An Essay on the Organization of Experience. Cambridge: Harvard University Press.

[B25] Gupta K. (2015). Compulsory sexuality: evaluating an emerging concept. Signs 41, 131–154. 10.1086/68177415801878

[B26] Hammersley M. Atkinson P. (1983). Ethnography: Principles in Practice. London: Tavistock.

[B27] Harding T. North N. Perkins R. (2008). Sexualizing men's touch: male nurses and the use of intimate touch in clinical practice. Res. Theory Nurs. Pract. 22, 88–102. 10.1891/1541-6577.22.2.88 18578219

[B28] Hine C. (2015). Ethnography for the Internet: Embedded, Embodied and Everyday. New York, NY: Routledge.

[B29] Hochschild A. R. (2003). The Commercialization of Intimate Life: Notes from Home and Work. Berkeley, CA: University of California Press.

[B30] Hochschild A. R. (2012). The Outsourced Self: What Happens When We Pay Others to Live Our Lives for Us. New York, NY: Picador.

[B31] Hollway W. (1984). Women's power in heterosexual sex. Women's Stud. Int. Forum 7, 63–68. 10.1016/0277-5395(84)90085-2

[B32] Honneth A. (1992). The Struggle for Recognition: The Moral Grammar of Social Conflicts. Cambridge: MIT Press.

[B33] Hooks B. (2000). Feminist Theory: From Margin to Center. Cambridge: South End Press.

[B34] Illouz E. (2007). Cold Intimacies: The Making of Emotional Capitalism. Cambridge: Polity Press.

[B35] Lupton B. (2000). Maintaining masculinity: men who do ‘women's work.' *Br. J. Manag*. 11, 33–48. 10.1111/1467-8551.11.s1.4

[B36] Marcus G. (1995). Ethnography in/of the world system: the emergence of multi-sited ethnography. Ann. Rev. Anthropol. 24, 95–117. 10.1146/annurev.an.24.100195.000523

[B37] Mayr C. (2023). Touch me if you can: intimate bodies at cuddle parties. J. Contemp. Ethno. 52, 218–242. 10.1177/08912416221100581

[B38] McDowell L. (2009). Working Bodies: Interactive Service Employment and Workplace Identities. Oxford: Wiley-Blackwell.

[B39] Mead G. H. (1972). Philosophy of the Act. Chicago, CA: University of Chicago Press.

[B40] Nonhoff M. (2017). Discourse analysis as critique. Palgrave Commun. 3, 1–11. 10.1057/palcomms.2017.74

[B41] Oerton S. (2004). Bodywork boundaries: power, politics and professionalism in therapeutic massage. Gender Work Organ. 11, 544–565. 10.1111/j.1468-0432.2004.00247.x

[B42] Oerton S. Phoenix J. (2001). Sex/bodywork: discourses and practices. Sexualities 4, 387–412. 10.1177/136346001004004001

[B43] Oliveira J. M. Costa C. Carneiro N. S. (2014). Troubling humanity: towards a queer feminist critical psychology. Ann. Rev. Crit. Psychol. 11, 41–58. Available online at: https://discourseunit.com/annual-review/11-2014/

[B44] Parsons T. (1951). The Social System. Chicago, CA: Free Press.

[B45] Przybylo E. (2011). Crisis and safety: the asexual in sexusociety. Sexualities 14, 444–461. 10.1177/1363460711406461

[B46] Przybylo E. (2014). Masculine Doubt and Sexual Wonder: Asexually-Identified Men Talk About Their (A)sexualities. in Asexualities: Feminist and Queer Perspectives. New York, NY: Routledge (pp. 225–246).

[B47] Rawls J. (1971). A Theory of Justice. Cambridge: Harvard University Pres

[B48] Reinharz S. Davidman L. (1992). Feminist Methods in Social Research. New York, NY: Oxford University Press.

[B49] Richard M. E. O'Sullivan L. F. Peppard T. (2020). Sexual harassment of massage therapists by their clients. Can. J. Human Sex. 29, 205–212. 10.3138/cjhs.2020-001136866180

[B50] Rosenblum N. (1998). Membership and Morals. Princeton, NJ: Princeton University Press.

[B51] Rubin G. (1984). “Thinking Sex: Notes for a Radical Theory of the Politics of Sexuality,” in Pleasure and Danger: Exploring Female Sexuality, ed C. Vance (Routledge and Kegan Paul) (pp. 267–319).

[B52] Sanders T. (2007). The politics of sexual citizenship: commercial sex and disability. Disabil. Soc. 22, 439–455. 10.1080/0968759070142747937300899

[B53] Sanders T. (2008). Paying for Pleasure: Men Who Buy Sex. Devon: Willan Publishing.

[B54] Sargent P. (2000). Real men or real teachers? Contradictions in the lives of men elementary teachers. Men Mascul. 2, 410–433. 10.1177/1097184X00002004003

[B55] Schütz A. (1944). The stranger: an essay in social psychology. Am. J. Sociol. 49, 499–507. 10.1086/219472

[B56] Schütz A. (1962). Collected Papers I: The Problem of Social Reality. The Hague: Martinus Nijhoff.

[B57] Seidler J. (1974). On using informants: a technique for collecting quantitative data and controlling measurement error in organization analysis. Am. Sociol. Rev. 39, 816–831. 10.2307/2094155

[B58] Simpson R. (2009). Men in Caring Occupations: Doing Gender Differently. Basingstoke: Palgrave Macmillan.

[B59] Simpson R. Slutskaya N. Lewis P. Höpfl H. (Eds.). (2012). Dirty Work: Concepts and Identities. Basingstoke: Palgrave Macmillan. 10.1057/9780230393530

[B60] Snow D. Rochford E. Worden S. Benford R. (1986). Frame alignment processes, micromobilization, and movement participation. Am. Sociol. Rev. 51, 464–481. 10.2307/2095581

[B61] Strauss A. (1978). “A social world perspective,” in Studies in Symbolic Interaction, ed N. Denzin (1, 119–128).

[B62] Sullivan K. (2012). Producing professionals: exploring gendered and embodied responses to practicing on the margins. Ephemera 12, 273–293.

[B63] Sullivan K. (2014). With(out) pleasure: desexualization, gender and sexuality at work. Organization 21, 346–364. 10.1177/1350508413519765

[B64] Szegheo-Lang N. de. (2015). Non-sexual spooning and inanimate affections: diversifying intimate knowledge. Atlantis Crit. Stud. Gender Cult. Soc. Justice 37, 14–22.

[B65] Thomas W. I. Thomas D. S. (1928). The Child in America: Behavior Problems and Programs. New York, NY: Knopf.

[B66] Thomas W. I. Znaniecki F. (1927). The Polish Peasant in Europe and America. New York, NY: Knopf.

[B67] Tremblay M-. A. (1957). The Key Informant Technique: A Nonethnographic Application. American Anthropologist, 59, 688–701. 10.1525/aa.1957.59.4.02a00100

[B68] Warren M. (2001). Democracy and Association. Princeton, NJ: Princeton University Press.

[B69] Wolkowitz C. (2002). The social relations of body work. Work Emp. Soc. 16, 497–510. 10.1177/095001702762217452

[B70] Zelizer V. (2005). The Purchase of Intimacy. Princeton, NJ: Princeton University Press.

